# Physical activity interventions for Asian Americans in cancer prevention and control: a scoping review

**DOI:** 10.1007/s10552-025-02104-1

**Published:** 2025-12-27

**Authors:** Jingxi Sheng, Haocen Wang, Mary Hitchcock

**Affiliations:** 1 School of Nursing, University of Wisconsin-Madison, 3141 Signe Skott Copper Hall 701 Highland Avenue Madison, Madison, WI 53705 USA; 2 School of Nursing, Purdue University, West Lafayette, IN USA; 3 University of Wisconsin-Madison, Ebling Library, Madison, WI USA

**Keywords:** Exercise, Minority, Survivorship, Oncology, Prevention

## Abstract

Despite the well-documented benefits of physical activity (PA) for cancer prevention and control, most PA interventions have not been tailored for Asian Americans (AsA), who face rising cancer incidence and a slower decrease in cancer mortality compared to other racial groups. This study aims to systematically review the PA interventions designed for AsA to prevent or manage cancer outcomes. A systematic search of five databases identified interventional studies with PA-promoting components in Asian American adults for cancer prevention or control, published before June 27, 2025, following the Cochrane Collaboration’s PRISMA guidelines. The systematic review identified 860 articles, with five studies meeting inclusion criteria, including four focused on cancer prevention and one on cancer control. Study populations included Filipinos (n = 2), Chinese (n = 1), Koreans (n = 1), and a mixed Asian and Hispanic group (n = 1). Studies were conducted in California (n = 2), Texas (n = 2), and New York (n = 1), with sample sizes ranging from 45 to 1,054 participants and program durations of 8 weeks to 18 months. Only one study exclusively targeted PA promotion; others included components like cancer screening and dietary interventions. All studies incorporated cultural adaptations, primarily language, while some featured culturally specific exercises and social apps. PA was self-reported in all studies, showing improvements post-intervention but limiting accuracy. These findings highlight the potential of PA interventions for AsA in cancer prevention and control, though their urban focus raises concerns about rural accessibility. Future efforts should prioritize rural outreach, enhance cultural adaptations, and assess long-term effectiveness and sustainability.

**Supplementary Information:**

The online version contains supplementary material available at 10.1007/s10552-025-02104-1.

## Introduction

Regular physical activity (PA) is well-established as a protective factor in the prevention of several types of cancer and as a supportive intervention in cancer control [1–3]. According to the National Cancer Institute and other research, PA is most notably associated with a reduced risk of the following cancers: breast, colon, endometrial, gastric, rectal, bladder, esophageal, liver, lung, kidney, blood, head and neck [3, 4]. Importantly, these protective effects are observed even after adjusting for factors like body weight, suggesting that PA may lower cancer risk through mechanisms beyond just weight control, such as improving immune function, reducing inflammation, and regulating hormone levels [3, 5, 6].

Cancer is the leading cause of death among Asian Americans (AsA), surpassing heart disease and other chronic illnesses [7, 8]. Despite being the fastest-growing racial group in the United States (US), AsA are often underrepresented in cancer prevention and control research. For example, A systematic review of cancer-related randomized controlled trials revealed that the median enrollment of AsA was only 1.4% compared to 88.7% of Non-Hispanic White, even though AsA comprise approximately 7% of the US population [9]. This underrepresentation poses significant challenges to addressing their unique cultural, behavioral, and environmental determinants of health. National guidelines and American Cancer Society recommend at least 150 min of moderate-to-vigorous PA (MVPA) weekly [10]. However, research has continuously shown that AsA are less likely to meet the PA recommendations than other racial groups [11–13]. Despite the low level of PA in this population, the development and implementation of culturally tailored PA interventions specific to AsA have been limited.

The purpose of this scoping review was to systematically map and describe existing PA interventions aimed at cancer prevention and control among AsA. Our aims are to summarize the current scope of evidence, identify gaps in research and practice, and inform directions for future culturally tailored interventions.

## Methods

A scoping review is designed to explore and map the breadth of literature on a given topic, identify knowledge gaps, and provide implications for future practice, research, and health policy [14, 15]. Given the limited number of studies conducted among AsA and the exploratory nature of our inquiry, we chose to conduct a scoping review rather than a systematic review [16]. We followed a five-stage framework proposed by Arksey and O’Malley [17], which includes: (1) identify the research questions, (2) identify relevant studies, (3) study selection, (4) charting the data, and (5) collating, summarizing and reporting the results. This review was guided by the Preferred Reporting Items for Systematic Reviews and Meta-Analyses extension for Scoping Review (PRISMA-ScR) [18].

### Search strategy

Five electronic databases were searched on August 8, 2024, and an updated search was conducted on June 27, 2025: PsycINFO, PubMed, CINAHL, Web of Science, and Scopus. The search was drafted by the first author (J.S.) and an expert in library sciences (M.H.). Concepts used in the search included “Asian Americans”, “Cancer”, and “physical activity”. The full search strategy is presented in Supplement A.

### Study selection

The eligibility criteria guiding the selection of research studies were as follows: (1) Empirical studies, including qualitative, quantitative, and mixed-methods studies or secondary analyses, that applied cancer prevention or control with PA components; (2) Descriptive studies that describe PA interventions for cancer prevention and control, development processes, and protocols; (3) Studies consisted of AsA adults aged 18 years or older; (4) Studies published in peer-reviewed English journals. Studies were excluded if they (1) included a PA component but not for cancer prevention and control; (2) included different racial and ethnic groups without separate reporting Asian Americans, (3) focused on Pacific Islanders; or (4) were books, government document, editorials, reviews, commentaries, or policy papers.

Citations were imported into EndNote and manually screened for duplicates by a librarian (MH). After duplicate removal, the remaining citations were uploaded to Covidence for title/abstract and full-text screening. Two reviewers (JS and HW) independently reviewed all records using Covidence and reached consensus on study inclusion through discussions.

### Data extraction

The following information was extracted from each included study: author(s), year, sample size, study population, study location, study design, type of cancer, purpose, program description, intervention length, and delivery modality. Two reviewers independently verified the table for accuracy. Key components related to PA were identified and categorized. These categories were developed iteratively through reviewer consensus. Table 1 presents the detailed study characteristics and PA components.

**Table 1:** Summary of included studies

Author (year)	Sample	Location	Design	Purpose	Program	Program component	Delivery modality
Seo et al. (2022)	n = 30, Korean	NYC, NY	Quasi-experimental trail	Breast cancer prevention	24-weeks educational program, including 8 weeks of in-class group session and 16 weeks follow-up	● Healthy weight ● Physically active lifestyle ● Healthy diet ● Breast cancer screening and adherence	In-person and online • Workshop • Snacks tasting • PA classes • Meal plan application • Group discussion • App, phone call & message follow-up
Deng (2019)	n = 50, Chinese	Houston, TX	Quasi-experimental trail (Pilot)	Cancer control (multiple types of cancer)	Home-based educational program 50-week	● Physical activity ● Diet	Home-based • Workbook • Newsletters • Consultation sessions • Telephone counseling
Raber (2018)	n = 1054, Hispanic (613) & Asian (332)	Houston, TX	Quasi-experimental trail	Colorectal cancer prevention	Educational program 3-week series, partnered with community sites	3 workshops ● Colon cancer ● Physical activity ● Diet	On site in person • Workshop • Cooking demonstrations • PA classes • Group discussion
Dirige (2013)	n = 673, Filipino	San Diego, CA	Cluster randomized control trial	Cancer prevention	Educational program 14-week, Trained leaders from each organization are then tasked with delivering the program	Intervention ● Physical activity ● Diet Control ● Cancer screening ● Alternative medicine ● Stress management	On site in person • Workshop • Cooking demonstrations • Recipe contents • Supermarket tours • PA classes
Maxwell (2002)	N = 530, Filipino	LA county, CA	Randomized control trial	Cancer prevention	Educational program	Intervention ● Cancer screening Control ● Physical activity	On site in person • Workshop • Various pictures of stretching and strengthening exercise • PA classes

## Results

**Fig. 1:**
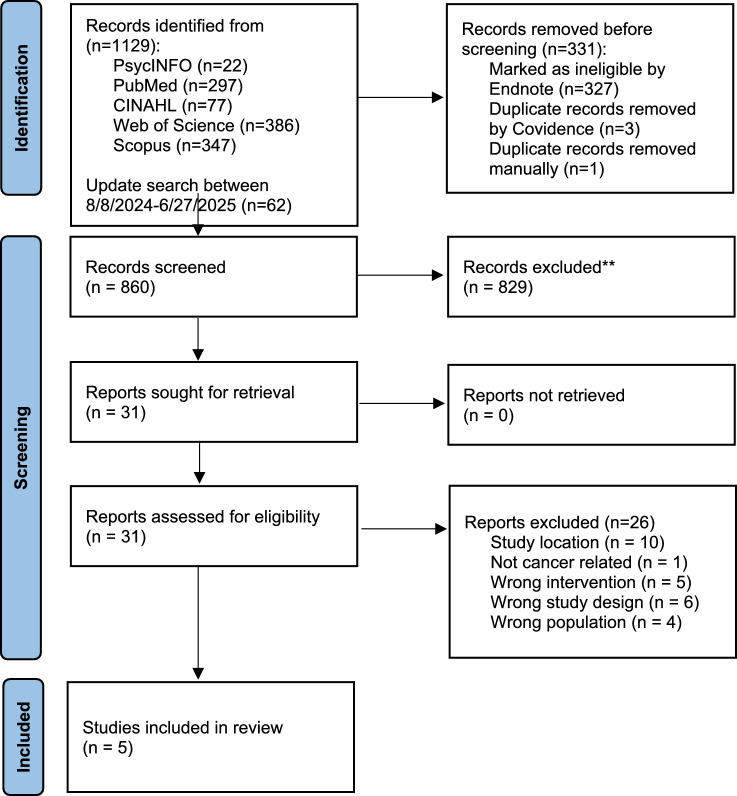
PRISMA flow diagram of the review

The initial search resulted in 860 citations after removing 331 duplicates. Among these, 829 studies were excluded after title and abstract screening. The remaining 31 studies were identified for full-text review. Five studies were included in this scoping review. A PRISMA flow diagram illustrates the study selection process (Fig. 1).

### Study characteristics

Among the five included studies, four focused on cancer prevention [19–22], and one focused on cancer control [23]. Two studies were randomized controlled trials [21, 22], three used quasi-experimental pre- and post-designs [19, 20, 23]. Studies were conducted in California (n = 2), Texas (n = 2), and New York (n = 1). Targeted populations included Filipinos (n = 2) [21, 22], Chinese (n = 1) [23], Koreans (n = 1) [19], and a mixed population including Asian and Hispanic groups (n = 1) [20], and sample sizes ranged from 30 to 770 participants. Four included studies reported participant-level program outcomes, with one providing qualitative data [19, 21–23], while one described program overview without reporting behavioral or health outcomes [20]. No study focused exclusively on targeting PA; all included additional components such as cancer screening [19–22], weight management [19], and dietary interventions [19–21, 23]. PA was often embedded within multi-component cancer prevention programs [19–21, 23] or used as a comparator in studies focused on screening [22]. In some cases, PA was part of a broader cancer prevention and control program [19–21, 23], while in others, it was used as a control condition compared to interventions focused on cancer screening [22].

Across studies, participants were predominantly mid-to-older adults, with a mean age ranging from 44.9 years to 63.2 years. Most were female (62.6—85.7%), married (68.1-85.7%), and had completed at least a high school education (52.6-89.3%). All studies included primarily foreign-born participants, with reported average US residency between 22 and 25 years in studies that included this data [21–23]. Interventionists included bilingual health educators [19, 20], registered dietitians [23], peer health committee members [21], or culturally matched physicians and nurses [22]. Retention rates were high across studies, ranging from 78.5% [21] to over 90% [19, 22, 23].

Three studies reported theoretical guidance: Social Cognitive Theory [23], Health Belief Model and PRECEDE–PROCEED [19], Indigenous and Adherence Models [22], while two did not specify a theoretical framework [20, 21].

### Settings

All included studies were conducted in urban community settings, such as churches, ethnic organizations, or local clinics. One study transitioned to an online format due to the COVID-19 pandemic [19]. None of the studies were conducted in rural areas or non-metropolitan areas.

### Physical activity (PA) components

We provide a brief narrative summary of each intervention’s PA component is provided in Table 2, including details on delivering personnel, PA measurements, and primary PA-related results. The PA components varied by intensity, structure, delivery mode, and behavioral emphasis across studies but generally fell into two categories: brief exposure-based approaches and structured, multi-session programs.

**Table 2:** Summary of physical activity (PA) components

Author (year)	Provider /Facilitator	Intervention description & components	Measures	Outcomes
Seo et al. (2022)	Personal trainer	The intervention consisted of two sections: one focusing on aerobic PA and the other on muscle-strengthening activities. Educational content covered: • How to calculate BMI and BMI categories • PA guidelines • Different PA types • Things to consider when making a personal PA plan • Health benefits of PA • Weight management	Self-reported program evaluation, qualitative interviews	• 100% reported that they increased their PA and reduced sitting time
Deng (2019)	Trained nonprofit organization staffs and volunteers	Participants were encouraged to walk at least 30 min per day. The walking component was supported through regular phone counseling, which aimed to enhance social support and self-efficacy, monitor progress, and identify and address barriers to maintaining regular PA	Self-reported using the Community Healthy Activities Model Program for Seniors	• 10% increasing of total participants walked at least 30 min/day • Frequency and caloric expenditure of moderate or higher intensity PA increased but not statistically significant
Raber (2018)	Certified Tai Chi instructors	Participants attended PA classes that included both demonstration and guided participation. Each session lasted 30–45 min Tai Chi or Qigong	n/a	n/a
Dirige (2013)	Experts	Guest lecturing sessions provided educational content and structured PA. Session's frequency varying by location. Topics included: • How to start a walking program • PA guidelines PA activities varied by session and included examples such as: Group aerobic classes, Kickboxing, dancing, gardening, basketball tournament	Self-reported Godin-Shephard PA survey	• PA group advanced their stage of change more in PA than cancer education group (p < 0.01) • At 18-month follow-up, PA group participants were more likely to meet PA guidelines (p < 0.001) and had a higher PA score (p = 0.02) compared to cancer education group
Maxwell (2002)	Nurses and physical therapist	One 60–90 min session exercise program, based on the 1996 Surgeon General’s Report, emphasized aerobic, strengthening, and flexibility exercises, with a focus on sustainability and enjoyment. Culturally tailored activities like dancing to Filipino music were included to increase engagement. Participants practiced stretching, brisk walking, and home-based strengthening exercises (e.g., lifting canned goods). The educator used visual aids, led demonstrations, encouraged group discussion, and provided individual feedback. Recommended frequency: aerobic and strengthening exercises 3x/week, stretching 3–5x/week. Handouts are provided to reinforce key messages. Sessions ended with informal discussions and refreshments	Baseline: two self-reported questions: whether participated in regular PA and frequency of exercise per week At 3 months: exercise assessment tool used in National Health and Nutrition Examination Survey II	• At 3 months post-intervention, the most commonly reported activities were walking, stretching, dancing, and gardening/yard work • Stretching was significantly more frequent among women in the exercise group compared to the screening group • Women in the exercise group also had a significantly higher total exercise score, indicating a potential intervention effect • Within the exercise group, attendees had notably higher scores than non-attendees (50.6 vs. 33.5, p < 0.01)

*PA* physical activity

The brief interventions introduced participants to PA through short, culturally relevant sessions [20, 22]. Maxwell et al.’s single-session workshop included demonstrations of culturally tailored aerobic, strengthening, and stretching exercises using household items, with an emphasis on safety and enjoyment [22]. Similarly, Raber et al. offered a one-time, 30–45-min exercise class adapted to participants’ cultural backgrounds (e.g., tai-chi, qi-gong), but provided no follow-up or behavior maintenance support [20].

In contrast, the structured interventions included ongoing PA promotion over time and integrated behavioral strategies to support sustained engagement [19, 21, 23]. Dirige et al.’s 18-month program featured monthly workshops with hands-on group activities (e.g., dancing, gardening, sports), designed to help participants meet moderate-to-vigorous PA guidelines [21]. Seo et al.’s 24-week intervention combined weekly educational sessions and three trainer-led exercise classes with 16 weeks of individual coaching via phone or text, aiming to build habits and reinforce self-efficacy [19]. This intervention also included content on breast cancer screening and diet, though the extent of PA-specific focus during follow-up coaching sessions was unclear. Moreover, Deng et al. implemented a 50-week home-based walking program supported by tailored print materials and 13 follow-up phone counseling sessions, but the program lacked supervised or group PA sessions [23].

### PA measures and outcomes

Among the included studies, Raber (2018) provided only a program overview without reporting details on measures and outcomes. The other four studies assessed changes in PA using self-reported data, with measurement tools varying across studies (Table 2). Two studies reported statistically significant improvements in PA outcomes [21, 22]. Participants in Dirige’s intervention group were more likely to meet MVPA guidelines (p < 0.001), had greater readiness for PA (p < 0.01), and reported higher overall PA scores (p = 0.02) compared to the control group. Maxwell found that PA participants had significantly higher total PA scores at 3-month follow-up compared to a cancer screening control group (p < 0.001). Seo’s participants qualitatively reported increased PA and reduced sitting time after the intervention [19]. In contrast, Deng found no significant changes in caloric expenditure (p = 0.818), frequency of moderate PA (p = 0.270), or walking ≥ 30 min per day (p = 0.096), despite the program’s long duration (50 weeks) and high retention [23]. Notably, all studies relied solely on self-reported PA outcomes using various tools to assess changes in PA behaviors.

### Culturally tailored intervention strategies

All five studies incorporated cultural tailoring strategies consistent with Kreuter et al.’s (2003) five-domain framework. Linguistic tailoring was implemented across all studies, with materials and sessions offered in targeted AsA subgroups (i.e., Chinese, Tagalog, Korean, and Vietnamese) [19–23]. Constituent-involving strategies, such as engaging community members or cultural experts in program development and delivery, were employed in four studies [19–21, 23]. Specifically, Dirige trained peer educators from Filipino-American organizations [21]; Maxwell engaged Filipino-born clinicians [22]; Deng’s materials were reviewed by community members [23]; and Seo’s program was developed with Korean cultural and content experts [19]. Socio-cultural strategies were also common, with interventions integrating commonly practiced PAs within the AsA community, which included tai-chi and qi-gong [20], dancing and gardening [21], or other culturally relevant exercises [19, 23]. Additionally, peripheral tailoring was explicitly noted in Raber’s program, which adapted visual materials to reflect the audience’s racial/ethnic background [20]. Evidential tailoring, presenting culturally relevant statistics or risk data, was reported in Seo (2023) and Raber (2018). However, no study empirically evaluated the independent effects of these tailoring strategies on PA outcomes.

## Discussion

This review systematically examines PA interventions for cancer prevention and control among AsA, aiming to assess the evidence base, identify research gaps, and inform future intervention strategies. The fact that only five studies met the inclusion criteria underscores a substantial gap in research focused on AsA in PA-related behavioral research for cancer prevention and control. Despite this gap, the reviewed interventions varied in design and scope, ranging from single-session educational exposures to multi-week, behaviorally grounded interventions. This heterogeneity likely contributed to the variation in PA outcomes and feasibility, emphasizing the importance of delivery structure, cultural relevance, and ongoing support in promoting sustained behavior change.

Depending on how studies measured and reported PA outcomes, it is difficult to draw a definitive conclusion about the overall effectiveness of included PA interventions on cancer prevention and control. Among the included studies, Raber (2018) provided only a program overview without reporting outcomes [20]. The remaining four studies assessed changes in PA using self-reported measures or qualitative descriptions with measurement tools varying across studies [19, 21–23]. Reliance on self-reported PA measures may limit the precision and comparability of these findings. Nevertheless, despite these differences and limitations associated with self-reported data, all four studies consistently indicated short-term increases in PA behaviors and readiness among AsA participants. These findings suggest that culturally tailored PA intervention can positively influence behaviors known to reduce cancer risk and improve survivorship. Overall, the available evidence indicates that such interventions are feasible and have the potential to support sustainable health-promoting behaviors in AsA populations when accessible.

Structured programs with different content modalities and behavioral strategies appeared more promising in building lasting habits. For example, Maxwell’s program includes only one 60–90 min session focused on physical activity; however, it features the most detailed, evidence-based content and was delivered by a physical therapist, ensuring high-quality expertise in program delivery [22]. Further, while some programs had longer durations and multiple follow-ups, such as Deng’s 50-week intervention, these often addressed multiple behaviors (e.g., cancer screening, nutrition) [23], making it unclear how much emphasis was placed specifically on PA. This, along with the exclusive use of self-reported measures and limited long-term outcome data, constrains conclusions about effectiveness. Encouragingly, all studies reported high retention rates, suggesting strong feasibility and acceptability of culturally tailored PA programs among the AsA population.

A key limitation identified across these interventions is the absence of objective measures and long-term follow-up to evaluate the sustainability and retention of PA behaviors. Although short-term improvements in activity levels and self-efficacy were reported, it remains unclear whether participants maintained these behaviors post-intervention, which is a significant concern given the well-documented challenge of sustaining PA after intervention cessation [24, 25]. To address this, future interventions should incorporate evidence-based strategies rooted in social cognitive theory [26], such as enhancing self-efficacy, self-control, and behavioral capability [27–29]. Practical behavioral strategies, such as breaking activities into manageable segments, exercising with a friend, incorporating rest periods, and using positive reinforcement, may help participants overcome barriers like fatigue and maintain regular activity [30, 31]. Furthermore, the use of objective tools (e.g., accelerometers or wearable activity monitors) would enhance the accuracy of PA measurement and strengthen conclusions about intervention effectiveness [32]. Lastly, extending follow-up assessments beyond the immediate post-intervention period is essential for evaluating the durability of behavior change, especially within culturally complex and underserved Asian communities.

To provide external context, PA and cancer intervention studies have been conducted in other racial and ethnic groups, as well as in broader populations. A quick literature search using the same strategies but without including the AsA concept yielded over 46,500 results on PubMed, compared to only 297 results when AsA were included, highlighting the relative scarcity of research in this group. This disparity indicates the urgent need to advance culturally tailored interventions that are relevant and accessible across different population groups. Evidence from broader populations underscores the benefits of PA for cancer outcomes. For example, a recent randomized controlled trial found that a 3-year structured exercise program initiated after adjuvant chemotherapy improved disease-free and overall survival, as well as physical function, in patients with stage III or high-risk stage II colon cancer [33]. Particularly, five-year disease-free survival was higher in the exercise group (80 vs. 74%; P = 0.017), and overall survival at 8 years was also improved significantly (90 vs. 83%; P = 0.022). Improvements in physical function were observed at 6 months and sustained through 24 months. Beyond colon cancer, evidence from other populations also supports the role of PA in cancer prevention and survivorship, demonstrating benefits such as quality of life, reduced fatigue, and lower mortality risk across several cancer types [2–4]. These consistent findings further reinforce the potential value of structured and culturally tailored PA interventions for AsA.

From a broader perspective, these findings highlight both opportunities and challenges for future inquiry. Moving forward, researchers should adopt the principles of community-based participatory research that prioritize equity, engaging community members not merely as participants but as active partners in co-creating culturally grounded and contextually meaningful interventions [34]. Further exploration is needed to understand how intersecting social determinants, such as gender norms, acculturation, caregiving responsibilities, and neighborhood environment, influence PA engagement, particularly among subgroups. Stakeholders may vary in their priorities: researchers may focus on measurable health outcomes, while communities may value aspects such as social bonding, stress relief, and cultural continuity, which have been observed in this population [35]. Community-based participatory research represents a powerful approach that connects scientific inquiry with real-world application by actively involving communities [36]. This approach is particularly critical for interventions that address health behaviors, which are deeply influenced by social and cultural contexts. Recognizing that individual behaviors are shaped by ongoing interactions with their environments, we advocate for a more integrated and community-centered approach to designing and evaluating interventions that prioritizes community perspectives, situational relevance, and the sustained well-being of participants.

### Limitations

This scoping review has several limitations that should be considered when interpreting the findings. First, the number of studies meeting the inclusion criteria was small, and many were pilot or exploratory in nature. All included studies were conducted in metropolitan areas, which restricts the generalizability and robustness of the conclusions. Second, there was considerable heterogeneity across studies regarding intervention design, outcome measures, and participant populations, which made direct comparisons and synthesis challenging. Third, all included studies relied solely on self-reported measures without objective validation, which may introduce reporting bias and limit accuracy. Future research with larger sample sizes, standardized outcome assessments, and rigorous methodologies is needed to strengthen the evidence base.

## Conclusion

This review underscores the early-stage and limited scope of research on PA interventions for cancer prevention and control among AsA. Of the five included studies, one was pilot in design, and one lacked outcome data altogether, leaving the current evidence base both narrow and preliminary. While the reviewed interventions demonstrated cultural responsiveness and strong participant retention, key methodological limitations, such as exclusive reliance on self-reported data, short follow-up, and unclear emphasis on PA in multi-component programs, hinder conclusions about their long-term effectiveness. Future research should prioritize rigorously designed, adequately powered trials that incorporate objective PA measurements, extended follow-up, and clear reporting. Isolating PA effects in multi-behavior interventions and evaluating cultural tailoring are also crucial. To ensure relevance and sustainability, interventions should be grounded in community-based participatory research frameworks that engage AsA communities as active partners. Such approaches are important to designing interventions that reflect the lived experiences, cultural contexts, and health priorities of diverse AsA populations, which help ensure both scientific rigor and real-world relevance.

## Supplementary Information

Below is the link to the electronic supplementary material.

Supplementary file1 (PDF 94 KB)

## Data Availability

No datasets were generated or analysed during the current study.
