# Supplementary material for: Physical activity interventions for Asian Americans in cancer prevention and control: a scoping review
Source: Cancer Causes Control. 2025 Dec 27;37(1):8. doi: 10.1007/s10552-025-02104-1 (PMC12743658; doi:10.1007/s10552-025-02104-1)
Supplement: Supplementary file 1 — Supplementary file1 (PDF 94 KB) [file 10552_2025_2104_MOESM1_ESM.pdf]

Search strategy

Database searched	Date searched	Results
APA PsycINFO (Ebsco)	8/8/2024	22
PubMed (MEDLINE)	8/8/2024	297
CINAHL (Ebsco)	8/8/2024	77
Web of Science (Clarivate)	8/8/2024	386
Scopus	8/8/2024	347
Total		1,129
Citations manually removed by Librarian in EndNote21	8/8/2024	327

Updated search (8/8/2024-6/27/2025): 62 items were found after duplicates were removed in Endnote

Exemplar article(s) provided by researcher (PMIDs): 31875686 (All items found in searches)

PubMed (updated search August 8, 2024)
("Asian"[Mesh] OR "Asian American Native Hawaiian and Pacific Islander"[Mesh]) OR "Asian People"[Mesh] OR "Asian people"[tiab] OR "Asian population"[tiab] OR "Chinese Americans"[tiab] OR "Asian Americans"[tiab:~3] OR "Asian American"[tiab:~2] OR ((immigrant*[tiab] OR emigrant*[tiab]) AND (Laotian[tiab] OR Filipino[tiab] OR Chinese[tiab] OR Japanese[tiab] OR Korean[tiab] OR Hmong[tiab] OR Malaysia[tiab] OR Nepal[tiab] OR Pakistan[tiab] OR Philippines[tiab] OR Taiwan[tiab] OR Singapore[tiab] OR Laos[tiab] OR Asian[tiab] OR Thai*[tiab] OR Vietnamese[tiab] OR Vietnam[tiab] OR Asian[tiab] OR Asia[tiab])) AND
("Neoplasms"[Mesh] OR "Cancer Survivors"[Mesh] OR "Radiation Oncology"[Mesh] OR cancer[tiab] OR tumor[tiab] OR tumour[tiab] OR malignan*[tiab] OR neoplasm[tiab] OR carcinoma[tiab] OR "cancer survivor"[tiab] OR "Cancer survivors"[tiab] OR oncology[tiab])
AND
(("Exercise"[Mesh] OR "Physical Fitness"[Mesh] OR "Exercise Movement Techniques"[Mesh] OR "Exercise Therapy"[Mesh] OR "Sports"[Mesh] OR walk*[tiab] OR exercise[tiab] OR exercising[tiab] OR "physical activit*"[tiab] OR "physically active"[tiab] OR "physical fitness"[tiab] OR sport*[tiab]))
297 results

PsycINFO
(((DE "Asians" OR DE "Central Asian Cultural Groups" OR DE "Chinese Cultural Groups" OR DE "Japanese Cultural Groups" OR DE "Japanese Americans" OR DE "Korean Cultural Groups" OR DE "Southeast Asian Cultural Groups" OR DE "Vietnamese Cultural Groups")) OR (American* N2 (Chinese OR Japanese OR Filipino OR Hmong OR Indochin* OR Thai OR

Lao OR Laotian OR Asian OR Vietnamese OR Korean))) OR (Asian* N2 (south OR Indian OR southeast OR east OR American)))
AND
(DE "Neoplasms" OR DE "Benign Neoplasms" OR DE "Breast Neoplasms" OR DE "Childhood Neoplasms" OR DE "Digestive System Neoplasms" OR DE "Endocrine Neoplasms" OR DE "Leukemias" OR DE "Lung Neoplasms" OR DE "Metastasis" OR DE "Nervous System Neoplasms" OR DE "Skin Neoplasms" OR DE "Terminal Cancer") OR TI (cancer OR neoplasm OR carcinoma OR sarcoma OR tumor OR tumour OR malignancy)
AND
(((((DE "Exercise" OR DE "Aerobic Exercise" OR DE "Weightlifting" OR DE "Yoga") OR (DE "Walking")) OR (DE "Physical Activity" OR DE "Physical Fitness" OR DE “Lifestyle”)) OR (DE "Active Living" OR DE "Activity Level" OR DE "Exercise Therapy")) OR (DE "Sports" OR DE "Athletic Participation" OR DE "Baseball" OR DE "Basketball" OR DE "College Sports" OR DE "Cycling" OR DE "Extreme Sports" OR DE "Football" OR DE "High School Sports" OR DE "Judo" OR DE "Martial Arts" OR DE "Soccer" OR DE "Swimming" OR DE "Tennis" OR DE "Sports (Attitudes Toward)")) OR TI (exercise OR exercising OR sports OR "physical activity" OR "physically active" OR "physical fitness")
22 results

CINAHL
((MH "Cancer Survivors") OR (MH "Neoplasms+") OR (MH "Carcinoma+") OR (MH "Sarcoma+") OR (MH "Hematologic Neoplasms+") OR (MH "Oncology+")) OR TI (cancer OR sarcoma OR neoplasm* OR oncology OR tumor)
AND
(MH "Asian Americans+") OR (MH "East Asian Americans+") OR (MH "South Asian Americans+") OR (MH "Southeast Asian Americans+") OR (MH "Asians+") OR (MH "East Asians+") OR (MH "South Asians+") OR (MH "Southeast Asians+") OR TI (Asian* N2 (south OR Indian OR southeast OR east OR American)) OR TI (American N2 (Hmong OR Japanese OR Korean OR Thai OR Taiwanese OR Chinese OR "Asian Indian*" OR Indochin* OR Vietnamese OR Asian))
AND
(MH "Exercise+") OR (MH "Therapeutic Exercise+") OR (MH "Aerobic Exercises+") OR (MH "Muscle Strengthening+") OR (MH "Upper Extremity Exercises+") OR (MH "Walking+") OR (MH "Physical Fitness+") OR (MH "Physical Activity") OR (MH "Sports") OR (MH "Exertion+")) OR TI (exercise OR exercising OR "physical activity" OR "physical activities" OR "physical fitness" OR "physically active" OR sports)
77 results

Asian Americans

- East Asian Americans
  - Chinese Americans
  - Hmong Americans
  - Japanese Americans
  - Korean Americans
  - Taiwanese Americans

- South Asian Americans
  - Asian Indian Americans
- Southeast Asian Americans
  - Cambodian Americans
  - Filipino Americans

Web of Science
(TS=(American NEAR/3 (Hmong OR Japanese OR Korean OR Asian OR Thai OR Taiwanese OR Chinese OR "Asian Indian*" OR Indochin* OR Vietnamese))) OR TS=(Asian* NEAR/3 (south OR Indian OR southeast OR east OR American))
AND
TS=(cancer OR carcinoma OR sarcoma OR neoplasm* OR oncology OR tumor OR tumour OR malignancy OR malignant)
AND
TS=(exercise OR exercising OR "physical activity" OR "physical activities" OR "physical fitness" OR "physically active" OR sports OR walking OR lifestyle)
386 results

Scopus

(TITLE-ABS-KEY (cancer OR carcinoma OR sarcoma OR neoplasm* OR oncology OR tumor OR tumour OR malignancy OR malignant))
AND
(TITLE-ABS-KEY (exercise OR exercising OR {physical activity} OR {physical activities} OR {physical fitness} OR {physically active} OR sports OR walking OR lifestyle))
AND
((TITLE-ABS-KEY (asian W/3 (hmong OR immigrant OR japanese OR korean OR thai OR taiwanese OR chinese OR "Asian Indian*" OR indochin* OR vietnamese)) OR TITLE-ABS-KEY (american W/3 (hmong OR japanese OR korean OR thai OR taiwanese OR chinese OR "Asian Indian*" OR indochin* OR vietnamese OR asian))))
347 results
